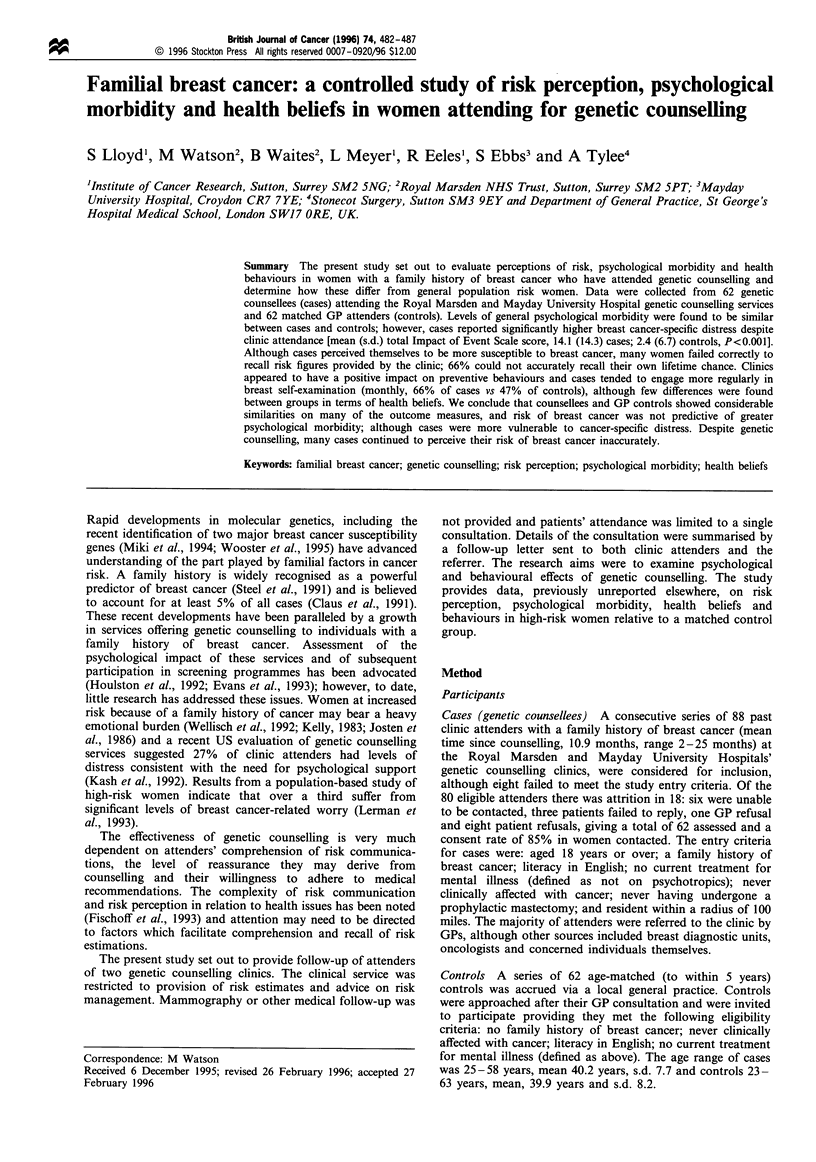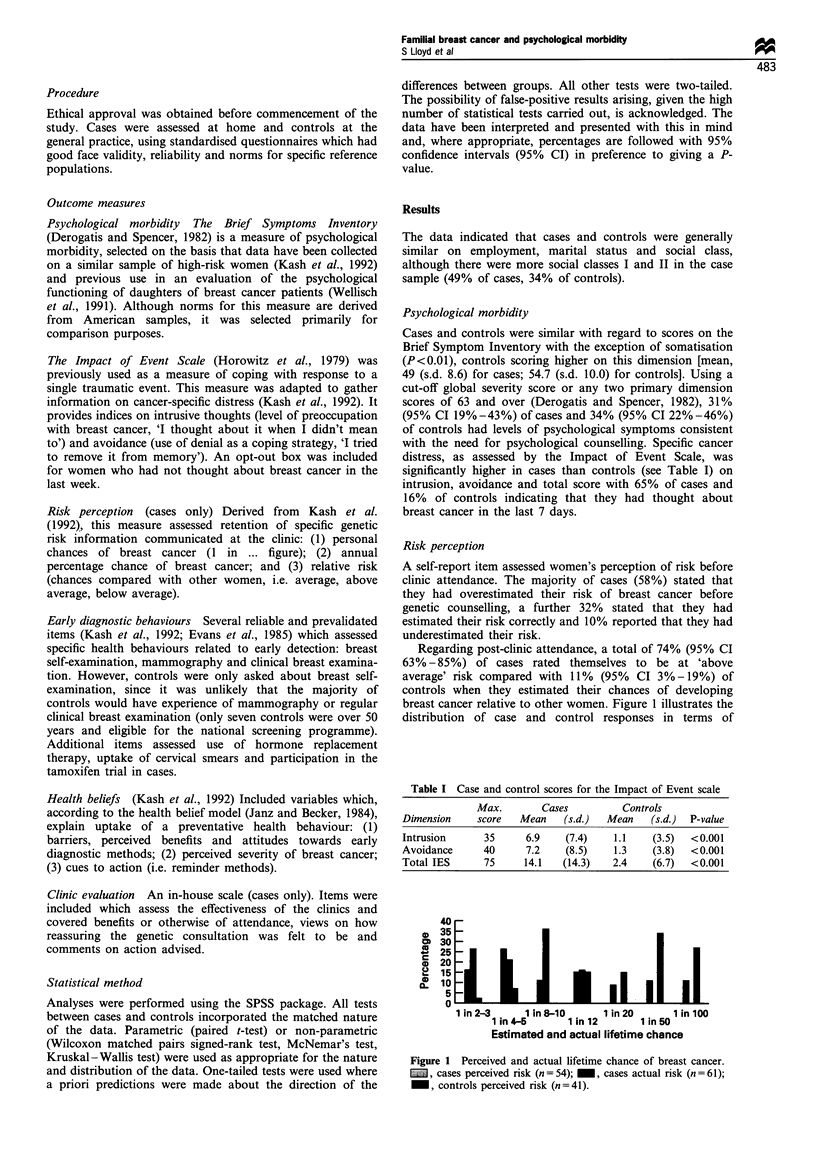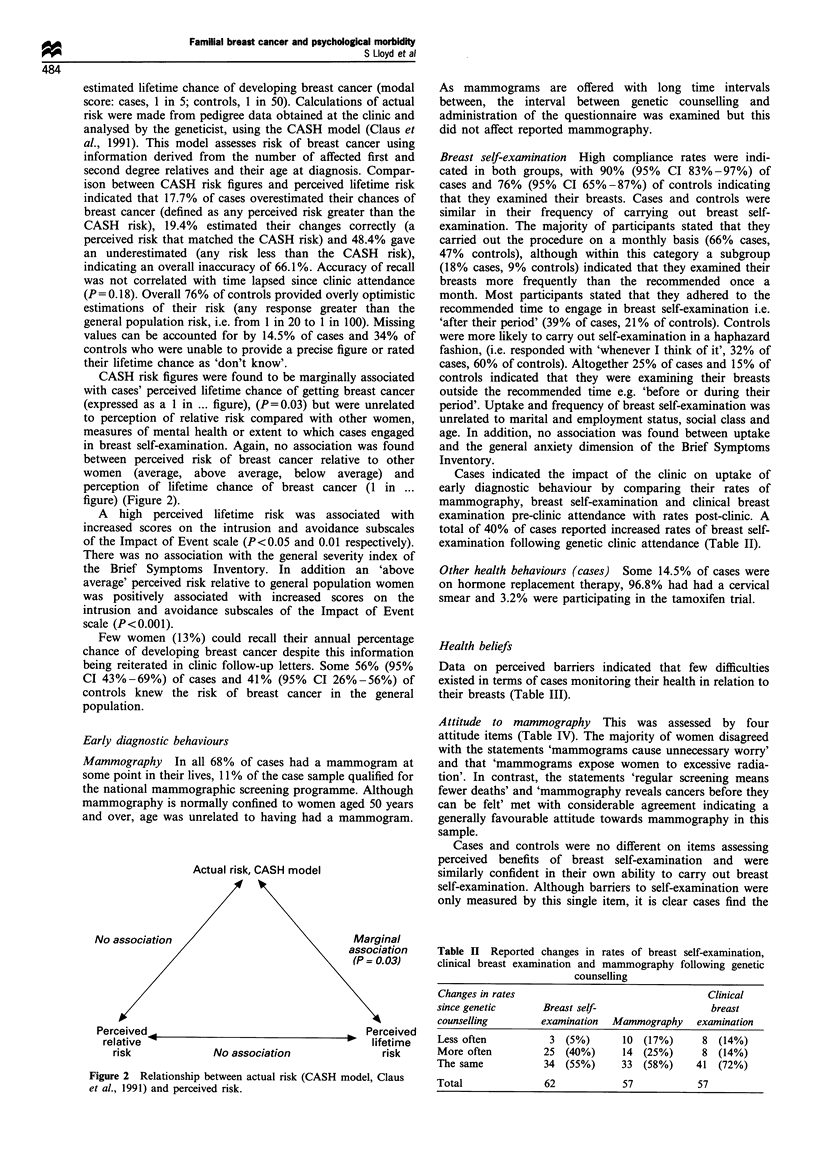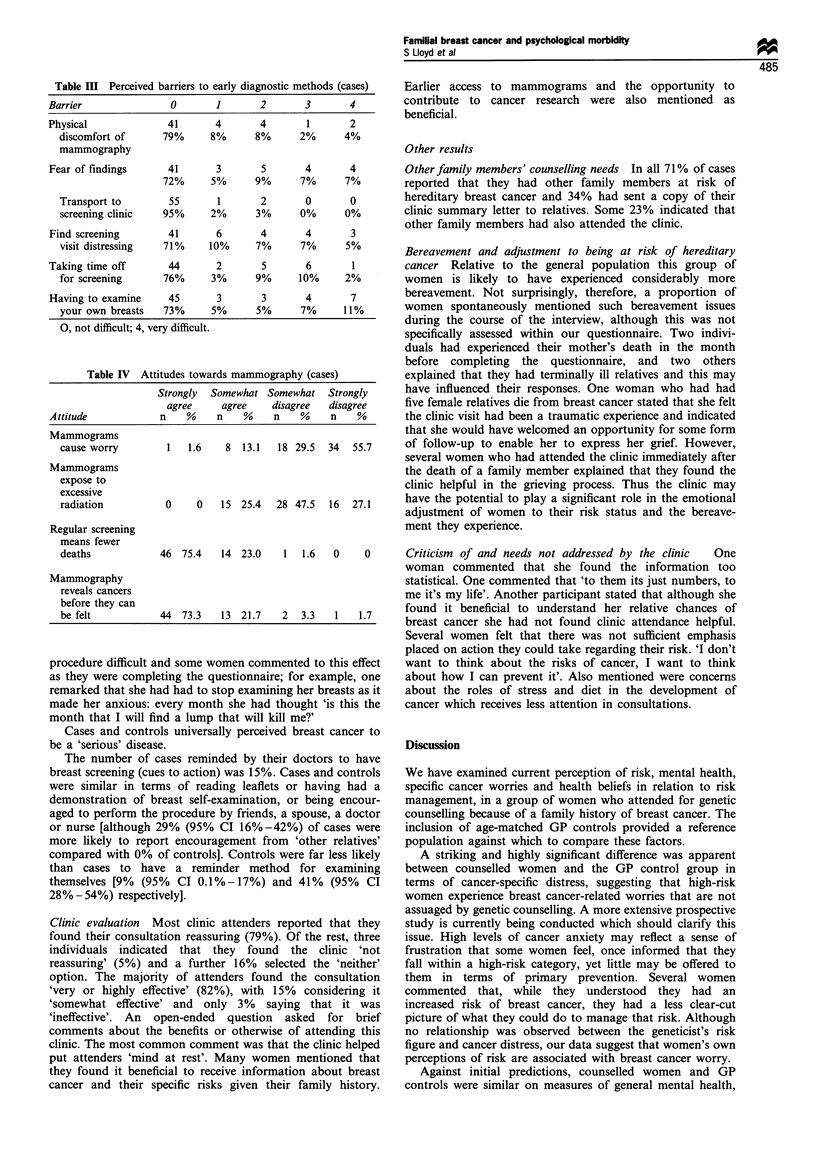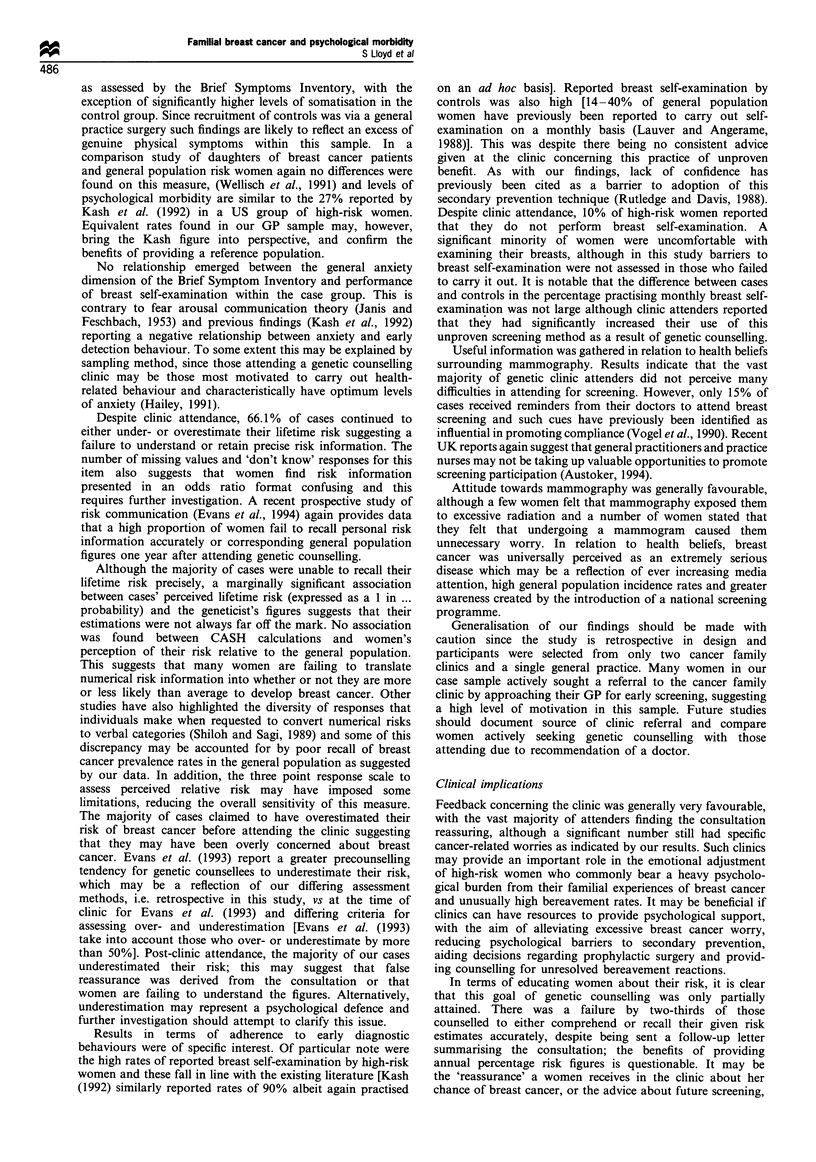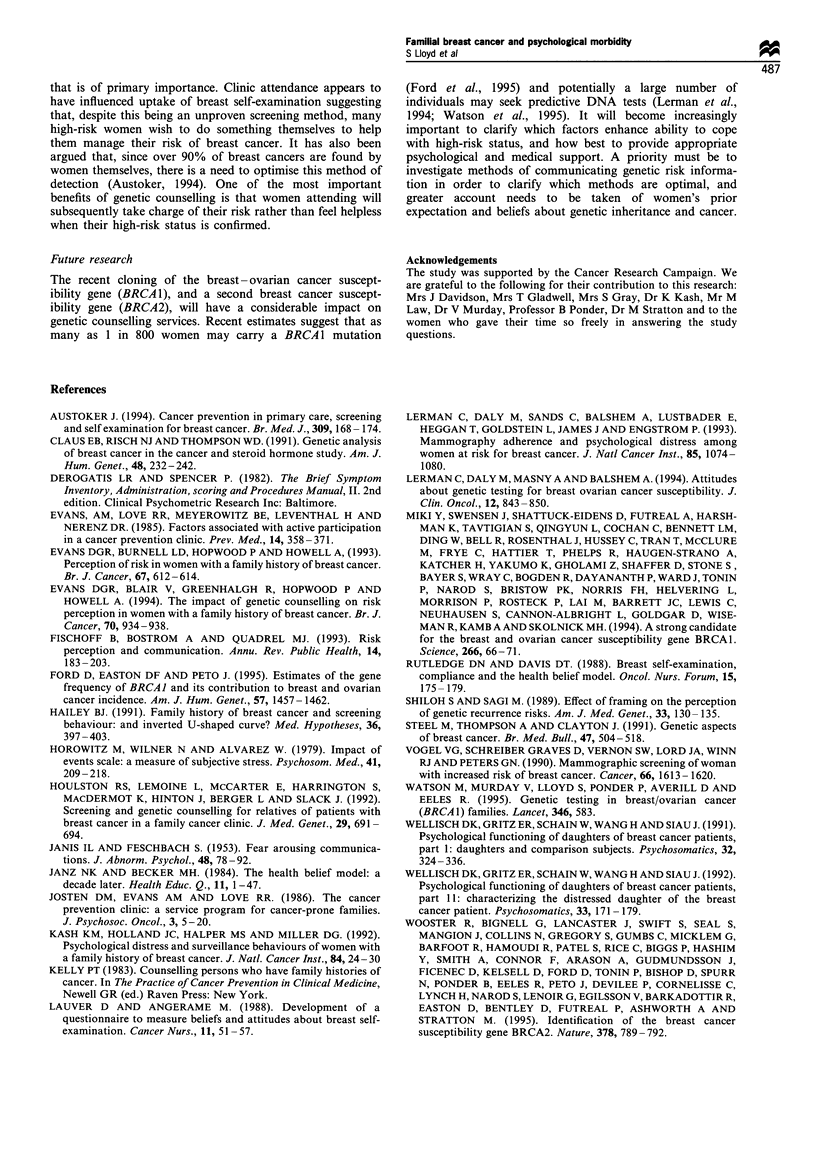# Familial breast cancer: a controlled study of risk perception, psychological morbidity and health beliefs in women attending for genetic counselling.

**DOI:** 10.1038/bjc.1996.387

**Published:** 1996-08

**Authors:** S. Lloyd, M. Watson, B. Waites, L. Meyer, R. Eeles, S. Ebbs, A. Tylee

**Affiliations:** Institute of Cancer Research, Sutton, Surrey, UK.

## Abstract

The present study set out to evaluate perceptions of risk, psychological morbidity and health behaviours in women with a family history of breast cancer who have attended genetic counselling and determine how these differ from general population risk women. Data were collected from 62 genetic counselees (cases) attending the Royal Marsden and Mayday University Hospital genetic counselling services and 62 matched GP attenders (controls). Levels of general psychological morbidity were found to be similar between cases and controls; however, cases reported significantly higher breast cancer-specific distress despite clinic attendance [mean (s.d.) total Impact of Event Scale score, 14.1 (14.3) cases; 2.4 (6.7) controls, P < 0.001]. Although cases perceived themselves to be more susceptible to breast cancer, many women failed correctly to recall risk figures provided by the clinic; 66% could not accurately recall their own lifetime chance. Clinics appeared to have a positive impact on preventive behaviours and cases tended to engage more regularly in breast self-examination (monthly, 66% of cases vs 47% of controls), although few differences were found between groups in terms of health beliefs. We conclude that counselees and GP controls showed considerable similarities on many of the outcome measures, and risk of breast cancer was not predictive of greater psychological morbidity; although cases were more vulnerable to cancer-specific distress. Despite genetic counselling, many cases continued to perceive their risk of breast cancer inaccurately.


British Journal of Cancer (1996) 74, 482-487
© 1996 Stockton Press All rights reserved 0007-0920/96 $12.00

### Familial breast cancer: a controlled study of risk perception, psychological morbidity and health beliefs in women attending for genetic counselling

S Lloyd[1], M Watson[2], B Waites[2], L Meyer[1], R Eeles[1], S Ebbs[3] and A Tylee[4]

[1]*Institute of Cancer Research, Sutton, Surrey SM2 5NG;* [2]*Royal Marsden NHS Trust, Sutton, Surrey SM2 5PT;* [3]*Mayday University Hospital, Croydon CR7 7YE;* [4]*Stonecot Surgery, Sutton SM3 9EY and Department of General Practice, St George's Hospital Medical School, London SW17 0RE, UK.*

**Summary** The present study set out to evaluate perceptions of risk, psychological morbidity and health behaviours in women with a family history of breast cancer who have attended genetic counselling and determine how these differ from general population risk women. Data were collected from 62 genetic counsellees (cases) attending the Royal Marsden and Mayday University Hospital genetic counselling services and 62 matched GP attenders (controls). Levels of general psychological morbidity were found to be similar between cases and controls; however, cases reported significantly higher breast cancer-specific distress despite clinic attendance [mean (s.d.) total Impact of Event Scale score, 14.1 (14.3) cases; 2.4 (6.7) controls, $P<0.001$]. Although cases perceived themselves to be more susceptible to breast cancer, many women failed correctly to recall risk figures provided by the clinic; 66% could not accurately recall their own lifetime chance. Clinics appeared to have a positive impact on preventive behaviours and cases tended to engage more regularly in breast self-examination (monthly, 66% of cases *vs* 47% of controls), although few differences were found between groups in terms of health beliefs. We conclude that counsellees and GP controls showed considerable similarities on many of the outcome measures, and risk of breast cancer was not predictive of greater psychological morbidity; although cases were more vulnerable to cancer-specific distress. Despite genetic counselling, many cases continued to perceive their risk of breast cancer inaccurately.

**Keywords:** familial breast cancer; genetic counselling; risk perception; psychological morbidity; health beliefs

Rapid developments in molecular genetics, including the recent identification of two major breast cancer susceptibility genes (Miki *et al.*, 1994; Wooster *et al.*, 1995) have advanced understanding of the part played by familial factors in cancer risk. A family history is widely recognised as a powerful predictor of breast cancer (Steel *et al.*, 1991) and is believed to account for at least 5% of all cases (Claus *et al.*, 1991). These recent developments have been paralleled by a growth in services offering genetic counselling to individuals with a family history of breast cancer. Assessment of the psychological impact of these services and of subsequent participation in screening programmes has been advocated (Houlston *et al.*, 1992; Evans *et al.*, 1993); however, to date, little research has addressed these issues. Women at increased risk because of a family history of cancer may bear a heavy emotional burden (Wellisch *et al.*, 1992; Kelly, 1983; Josten *et al.*, 1986) and a recent US evaluation of genetic counselling services suggested 27% of clinic attenders had levels of distress consistent with the need for psychological support (Kash *et al.*, 1992). Results from a population-based study of high-risk women indicate that over a third suffer from significant levels of breast cancer-related worry (Lerman *et al.*, 1993).

The effectiveness of genetic counselling is very much dependent on attenders' comprehension of risk communications, the level of reassurance they may derive from counselling and their willingness to adhere to medical recommendations. The complexity of risk communication and risk perception in relation to health issues has been noted (Fischoff *et al.*, 1993) and attention may need to be directed to factors which facilitate comprehension and recall of risk estimations.

The present study set out to provide follow-up of attenders of two genetic counselling clinics. The clinical service was restricted to provision of risk estimates and advice on risk management. Mammography or other medical follow-up was not provided and patients' attendance was limited to a single consultation. Details of the consultation were summarised by a follow-up letter sent to both clinic attenders and the referrer. The research aims were to examine psychological and behavioural effects of genetic counselling. The study provides data, previously unreported elsewhere, on risk perception, psychological morbidity, health beliefs and behaviours in high-risk women relative to a matched control group.

### Method

### Participants

*Cases (genetic counsellees)* A consecutive series of 88 past clinic attenders with a family history of breast cancer (mean time since counselling, 10.9 months, range 2–25 months) at the Royal Marsden and Mayday University Hospitals' genetic counselling clinics, were considered for inclusion, although eight failed to meet the study entry criteria. Of the 80 eligible attenders there was attrition in 18: six were unable to be contacted, three patients failed to reply, one GP refusal and eight patient refusals, giving a total of 62 assessed and a consent rate of 85% in women contacted. The entry criteria for cases were: aged 18 years or over; a family history of breast cancer; literacy in English; no current treatment for mental illness (defined as not on psychotropics); never clinically affected with cancer; never having undergone a prophylactic mastectomy; and resident within a radius of 100 miles. The majority of attenders were referred to the clinic by GPs, although other sources included breast diagnostic units, oncologists and concerned individuals themselves.

*Controls* A series of 62 age-matched (to within 5 years) controls was accrued via a local general practice. Controls were approached after their GP consultation and were invited to participate providing they met the following eligibility criteria: no family history of breast cancer; never clinically affected with cancer; literacy in English; no current treatment for mental illness (defined as above). The age range of cases was 25–58 years, mean 40.2 years, s.d. 7.7 and controls 23–63 years, mean, 39.9 years and s.d. 8.2.

Correspondence: M Watson
Received 6 December 1995; revised 26 February 1996; accepted 27 February 1996

### *Procedure*

Ethical approval was obtained before commencement of the study. Cases were assessed at home and controls at the general practice, using standardised questionnaires which had good face validity, reliability and norms for specific reference populations.

### *Outcome measures*

*Psychological morbidity The Brief Symptoms Inventory* (Derogatis and Spencer, 1982) is a measure of psychological morbidity, selected on the basis that data have been collected on a similar sample of high-risk women (Kash *et al.*, 1992) and previous use in an evaluation of the psychological functioning of daughters of breast cancer patients (Wellisch *et al.*, 1991). Although norms for this measure are derived from American samples, it was selected primarily for comparison purposes.

*The Impact of Event Scale* (Horowitz *et al.*, 1979) was previously used as a measure of coping with response to a single traumatic event. This measure was adapted to gather information on cancer-specific distress (Kash *et al.*, 1992). It provides indices on intrusive thoughts (level of preoccupation with breast cancer, 'I thought about it when I didn't mean to') and avoidance (use of denial as a coping strategy, 'I tried to remove it from memory'). An opt-out box was included for women who had not thought about breast cancer in the last week.

*Risk perception* (cases only) Derived from Kash *et al.* (1992), this measure assessed retention of specific genetic risk information communicated at the clinic: (1) personal chances of breast cancer (1 in ... figure); (2) annual percentage chance of breast cancer; and (3) relative risk (chances compared with other women, i.e. average, above average, below average).

*Early diagnostic behaviours* Several reliable and prevalidated items (Kash *et al.*, 1992; Evans *et al.*, 1985) which assessed specific health behaviours related to early detection: breast self-examination, mammography and clinical breast examination. However, controls were only asked about breast self-examination, since it was unlikely that the majority of controls would have experience of mammography or regular clinical breast examination (only seven controls were over 50 years and eligible for the national screening programme). Additional items assessed use of hormone replacement therapy, uptake of cervical smears and participation in the tamoxifen trial in cases.

*Health beliefs* (Kash *et al.*, 1992) Included variables which, according to the health belief model (Janz and Becker, 1984), explain uptake of a preventative health behaviour: (1) barriers, perceived benefits and attitudes towards early diagnostic methods; (2) perceived severity of breast cancer; (3) cues to action (i.e. reminder methods).

*Clinic evaluation* An in-house scale (cases only). Items were included which assess the effectiveness of the clinics and covered benefits or otherwise of attendance, views on how reassuring the genetic consultation was felt to be and comments on action advised.

### *Statistical method*

Analyses were performed using the SPSS package. All tests between cases and controls incorporated the matched nature of the data. Parametric (paired *t*-test) or non-parametric (Wilcoxon matched pairs signed-rank test, McNemar's test, Kruskal–Wallis test) were used as appropriate for the nature and distribution of the data. One-tailed tests were used where a priori predictions were made about the direction of the differences between groups. All other tests were two-tailed. The possibility of false-positive results arising, given the high number of statistical tests carried out, is acknowledged. The data have been interpreted and presented with this in mind and, where appropriate, percentages are followed with 95% confidence intervals (95% CI) in preference to giving a *P*-value.

### Results

The data indicated that cases and controls were generally similar on employment, marital status and social class, although there were more social classes I and II in the case sample (49% of cases, 34% of controls).

### *Psychological morbidity*

Cases and controls were similar with regard to scores on the Brief Symptom Inventory with the exception of somatisation ($P<0.01$), controls scoring higher on this dimension [mean, 49 (s.d. 8.6) for cases; 54.7 (s.d. 10.0) for controls]. Using a cut-off global severity score or any two primary dimension scores of 63 and over (Derogatis and Spencer, 1982), 31% (95% CI 19%–43%) of cases and 34% (95% CI 22%–46%) of controls had levels of psychological symptoms consistent with the need for psychological counselling. Specific cancer distress, as assessed by the Impact of Event Scale, was significantly higher in cases than controls (see Table I) on intrusion, avoidance and total score with 65% of cases and 16% of controls indicating that they had thought about breast cancer in the last 7 days.

### *Risk perception*

A self-report item assessed women's perception of risk before clinic attendance. The majority of cases (58%) stated that they had overestimated their risk of breast cancer before genetic counselling, a further 32% stated that they had estimated their risk correctly and 10% reported that they had underestimated their risk.

Regarding post-clinic attendance, a total of 74% (95% CI 63%–85%) of cases rated themselves to be at 'above average' risk compared with 11% (95% CI 3%–19%) of controls when they estimated their chances of developing breast cancer relative to other women. Figure 1 illustrates the distribution of case and control responses in terms of

**Table I** Case and control scores for the Impact of Event scale

| *Dimension* | *Max. score* | *Cases Mean* | *(s.d.)* | *Controls Mean* | *(s.d.)* | P-*value* |
|---|---|---|---|---|---|---|
| Intrusion | 35 | 6.9 | (7.4) | 1.1 | (3.5) | <0.001 |
| Avoidance | 40 | 7.2 | (8.5) | 1.3 | (3.8) | <0.001 |
| Total IES | 75 | 14.1 | (14.3) | 2.4 | (6.7) | <0.001 |

**Figure 1** Perceived and actual lifetime chance of breast cancer. ▒, cases perceived risk ($n=54$); ■, cases actual risk ($n=61$); ■, controls perceived risk ($n=41$).

estimated lifetime chance of developing breast cancer (modal score: cases, 1 in 5; controls, 1 in 50). Calculations of actual risk were made from pedigree data obtained at the clinic and analysed by the geneticist, using the CASH model (Claus *et al.*, 1991). This model assesses risk of breast cancer using information derived from the number of affected first and second degree relatives and their age at diagnosis. Comparison between CASH risk figures and perceived lifetime risk indicated that 17.7% of cases overestimated their chances of breast cancer (defined as any perceived risk greater than the CASH risk), 19.4% estimated their changes correctly (a perceived risk that matched the CASH risk) and 48.4% gave an underestimated (any risk less than the CASH risk), indicating an overall inaccuracy of 66.1%. Accuracy of recall was not correlated with time lapsed since clinic attendance ($P=0.18$). Overall 76% of controls provided overly optimistic estimations of their risk (any response greater than the general population risk, i.e. from 1 in 20 to 1 in 100). Missing values can be accounted for by 14.5% of cases and 34% of controls who were unable to provide a precise figure or rated their lifetime chance as 'don't know'.

CASH risk figures were found to be marginally associated with cases' perceived lifetime chance of getting breast cancer (expressed as a 1 in ... figure), ($P=0.03$) but were unrelated to perception of relative risk compared with other women, measures of mental health or extent to which cases engaged in breast self-examination. Again, no association was found between perceived risk of breast cancer relative to other women (average, above average, below average) and perception of lifetime chance of breast cancer (1 in ... figure) (Figure 2).

A high perceived lifetime risk was associated with increased scores on the intrusion and avoidance subscales of the Impact of Event scale ($P<0.05$ and 0.01 respectively). There was no association with the general severity index of the Brief Symptoms Inventory. In addition an 'above average' perceived risk relative to general population women was positively associated with increased scores on the intrusion and avoidance subscales of the Impact of Event scale ($P<0.001$).

Few women (13%) could recall their annual percentage chance of developing breast cancer despite this information being reiterated in clinic follow-up letters. Some 56% (95% CI 43%-69%) of cases and 41% (95% CI 26%-56%) of controls knew the risk of breast cancer in the general population.

### *Early diagnostic behaviours*

*Mammography* In all 68% of cases had a mammogram at some point in their lives, 11% of the case sample qualified for the national mammographic screening programme. Although mammography is normally confined to women aged 50 years and over, age was unrelated to having had a mammogram.

**Figure 2** Relationship between actual risk (CASH model, Claus *et al.*, 1991) and perceived risk.

As mammograms are offered with long time intervals between, the interval between genetic counselling and administration of the questionnaire was examined but this did not affect reported mammography.

*Breast self-examination* High compliance rates were indicated in both groups, with 90% (95% CI 83%-97%) of cases and 76% (95% CI 65%-87%) of controls indicating that they examined their breasts. Cases and controls were similar in their frequency of carrying out breast self-examination. The majority of participants stated that they carried out the procedure on a monthly basis (66% cases, 47% controls), although within this category a subgroup (18% cases, 9% controls) indicated that they examined their breasts more frequently than the recommended once a month. Most participants stated that they adhered to the recommended time to engage in breast self-examination i.e. 'after their period' (39% of cases, 21% of controls). Controls were more likely to carry out self-examination in a haphazard fashion, (i.e. responded with 'whenever I think of it', 32% of cases, 60% of controls). Altogether 25% of cases and 15% of controls indicated that they were examining their breasts outside the recommended time e.g. 'before or during their period'. Uptake and frequency of breast self-examination was unrelated to marital and employment status, social class and age. In addition, no association was found between uptake and the general anxiety dimension of the Brief Symptoms Inventory.

Cases indicated the impact of the clinic on uptake of early diagnostic behaviour by comparing their rates of mammography, breast self-examination and clinical breast examination pre-clinic attendance with rates post-clinic. A total of 40% of cases reported increased rates of breast self-examination following genetic clinic attendance (Table II).

*Other health behaviours (cases)* Some 14.5% of cases were on hormone replacement therapy, 96.8% had had a cervical smear and 3.2% were participating in the tamoxifen trial.

### *Health beliefs*

Data on perceived barriers indicated that few difficulties existed in terms of cases monitoring their health in relation to their breasts (Table III).

*Attitude to mammography* This was assessed by four attitude items (Table IV). The majority of women disagreed with the statements 'mammograms cause unnecessary worry' and that 'mammograms expose women to excessive radiation'. In contrast, the statements 'regular screening means fewer deaths' and 'mammography reveals cancers before they can be felt' met with considerable agreement indicating a generally favourable attitude towards mammography in this sample.

Cases and controls were no different on items assessing perceived benefits of breast self-examination and were similarly confident in their own ability to carry out breast self-examination. Although barriers to self-examination were only measured by this single item, it is clear cases find the

**Table II** Reported changes in rates of breast self-examination, clinical breast examination and mammography following genetic counselling

| *Changes in rates since genetic counselling* | *Breast self-examination* | *Mammography* | *Clinical breast examination* |
|---|---|---|---|
| Less often | 3 (5%) | 10 (17%) | 8 (14%) |
| More often | 25 (40%) | 14 (25%) | 8 (14%) |
| The same | 34 (55%) | 33 (58%) | 41 (72%) |
| Total | 62 | 57 | 57 |

**Table III** Perceived barriers to early diagnostic methods (cases)

| *Barrier* | *0* | *1* | *2* | *3* | *4* |
|---|---|---|---|---|---|
| Physical discomfort of mammography | 41<br>79% | 4<br>8% | 4<br>8% | 1<br>2% | 2<br>4% |
| Fear of findings | 41<br>72% | 3<br>5% | 5<br>9% | 4<br>7% | 4<br>7% |
| Transport to screening clinic | 55<br>95% | 1<br>2% | 2<br>3% | 0<br>0% | 0<br>0% |
| Find screening visit distressing | 41<br>71% | 6<br>10% | 4<br>7% | 4<br>7% | 3<br>5% |
| Taking time off for screening | 44<br>76% | 2<br>3% | 5<br>9% | 6<br>10% | 1<br>2% |
| Having to examine your own breasts | 45<br>73% | 3<br>5% | 3<br>5% | 4<br>7% | 7<br>11% |

O, not difficult; 4, very difficult.

**Table IV** Attitudes towards mammography (cases)

| *Attitude* | *Strongly agree* n | % | *Somewhat agree* n | % | *Somewhat disagree* n | % | *Strongly disagree* n | % |
|---|---|---|---|---|---|---|---|---|
| Mammograms cause worry | 1 | 1.6 | 8 | 13.1 | 18 | 29.5 | 34 | 55.7 |
| Mammograms expose to excessive radiation | 0 | 0 | 15 | 25.4 | 28 | 47.5 | 16 | 27.1 |
| Regular screening means fewer deaths | 46 | 75.4 | 14 | 23.0 | 1 | 1.6 | 0 | 0 |
| Mammography reveals cancers before they can be felt | 44 | 73.3 | 13 | 21.7 | 2 | 3.3 | 1 | 1.7 |

procedure difficult and some women commented to this effect as they were completing the questionnaire; for example, one remarked that she had had to stop examining her breasts as it made her anxious: every month she had thought 'is this the month that I will find a lump that will kill me?'

Cases and controls universally perceived breast cancer to be a 'serious' disease.

The number of cases reminded by their doctors to have breast screening (cues to action) was 15%. Cases and controls were similar in terms of reading leaflets or having had a demonstration of breast self-examination, or being encouraged to perform the procedure by friends, a spouse, a doctor or nurse [although 29% (95% CI 16%-42%) of cases were more likely to report encouragement from 'other relatives' compared with 0% of controls]. Controls were far less likely than cases to have a reminder method for examining themselves [9% (95% CI 0.1%-17%) and 41% (95% CI 28%-54%) respectively].

*Clinic evaluation* Most clinic attenders reported that they found their consultation reassuring (79%). Of the rest, three individuals indicated that they found the clinic 'not reassuring' (5%) and a further 16% selected the 'neither' option. The majority of attenders found the consultation 'very or highly effective' (82%), with 15% considering it 'somewhat effective' and only 3% saying that it was 'ineffective'. An open-ended question asked for brief comments about the benefits or otherwise of attending this clinic. The most common comment was that the clinic helped put attenders 'mind at rest'. Many women mentioned that they found it beneficial to receive information about breast cancer and their specific risks given their family history. Earlier access to mammograms and the opportunity to contribute to cancer research were also mentioned as beneficial.

*Other results*

*Other family members' counselling needs* In all 71% of cases reported that they had other family members at risk of hereditary breast cancer and 34% had sent a copy of their clinic summary letter to relatives. Some 23% indicated that other family members had also attended the clinic.

*Bereavement and adjustment to being at risk of hereditary cancer* Relative to the general population this group of women is likely to have experienced considerably more bereavement. Not surprisingly, therefore, a proportion of women spontaneously mentioned such bereavement issues during the course of the interview, although this was not specifically assessed within our questionnaire. Two individuals had experienced their mother's death in the month before completing the questionnaire, and two others explained that they had terminally ill relatives and this may have influenced their responses. One woman who had had five female relatives die from breast cancer stated that she felt the clinic visit had been a traumatic experience and indicated that she would have welcomed an opportunity for some form of follow-up to enable her to express her grief. However, several women who had attended the clinic immediately after the death of a family member explained that they found the clinic helpful in the grieving process. Thus the clinic may have the potential to play a significant role in the emotional adjustment of women to their risk status and the bereavement they experience.

*Criticism of and needs not addressed by the clinic* One woman commented that she found the information too statistical. One commented that 'to them its just numbers, to me it's my life'. Another participant stated that although she found it beneficial to understand her relative chances of breast cancer she had not found clinic attendance helpful. Several women felt that there was not sufficient emphasis placed on action they could take regarding their risk. 'I don't want to think about the risks of cancer, I want to think about how I can prevent it'. Also mentioned were concerns about the roles of stress and diet in the development of cancer which receives less attention in consultations.

### Discussion

We have examined current perception of risk, mental health, specific cancer worries and health beliefs in relation to risk management, in a group of women who attended for genetic counselling because of a family history of breast cancer. The inclusion of age-matched GP controls provided a reference population against which to compare these factors.

A striking and highly significant difference was apparent between counselled women and the GP control group in terms of cancer-specific distress, suggesting that high-risk women experience breast cancer-related worries that are not assuaged by genetic counselling. A more extensive prospective study is currently being conducted which should clarify this issue. High levels of cancer anxiety may reflect a sense of frustration that some women feel, once informed that they fall within a high-risk category, yet little may be offered to them in terms of primary prevention. Several women commented that, while they understood they had an increased risk of breast cancer, they had a less clear-cut picture of what they could do to manage that risk. Although no relationship was observed between the geneticist's risk figure and cancer distress, our data suggest that women's own perceptions of risk are associated with breast cancer worry.

Against initial predictions, counselled women and GP controls were similar on measures of general mental health, as assessed by the Brief Symptoms Inventory, with the exception of significantly higher levels of somatisation in the control group. Since recruitment of controls was via a general practice surgery such findings are likely to reflect an excess of genuine physical symptoms within this sample. In a comparison study of daughters of breast cancer patients and general population risk women again no differences were found on this measure, (Wellisch *et al.*, 1991) and levels of psychological morbidity are similar to the 27% reported by Kash *et al.* (1992) in a US group of high-risk women. Equivalent rates found in our GP sample may, however, bring the Kash figure into perspective, and confirm the benefits of providing a reference population.

No relationship emerged between the general anxiety dimension of the Brief Symptom Inventory and performance of breast self-examination within the case group. This is contrary to fear arousal communication theory (Janis and Feschbach, 1953) and previous findings (Kash *et al.*, 1992) reporting a negative relationship between anxiety and early detection behaviour. To some extent this may be explained by sampling method, since those attending a genetic counselling clinic may be those most motivated to carry out health-related behaviour and characteristically have optimum levels of anxiety (Hailey, 1991).

Despite clinic attendance, 66.1% of cases continued to either under- or overestimate their lifetime risk suggesting a failure to understand or retain precise risk information. The number of missing values and 'don't know' responses for this item also suggests that women find risk information presented in an odds ratio format confusing and this requires further investigation. A recent prospective study of risk communication (Evans *et al.*, 1994) again provides data that a high proportion of women fail to recall personal risk information accurately or corresponding general population figures one year after attending genetic counselling.

Although the majority of cases were unable to recall their lifetime risk precisely, a marginally significant association between cases' perceived lifetime risk (expressed as a 1 in ... probability) and the geneticist's figures suggests that their estimations were not always far off the mark. No association was found between CASH calculations and women's perception of their risk relative to the general population. This suggests that many women are failing to translate numerical risk information into whether or not they are more or less likely than average to develop breast cancer. Other studies have also highlighted the diversity of responses that individuals make when requested to convert numerical risks to verbal categories (Shiloh and Sagi, 1989) and some of this discrepancy may be accounted for by poor recall of breast cancer prevalence rates in the general population as suggested by our data. In addition, the three point response scale to assess perceived relative risk may have imposed some limitations, reducing the overall sensitivity of this measure. The majority of cases claimed to have overestimated their risk of breast cancer before attending the clinic suggesting that they may have been overly concerned about breast cancer. Evans *et al.* (1993) report a greater precounselling tendency for genetic counsellees to underestimate their risk, which may be a reflection of our differing assessment methods, i.e. retrospective in this study, *vs* at the time of clinic for Evans *et al.* (1993) and differing criteria for assessing over- and underestimation [Evans *et al.* (1993) take into account those who over- or underestimate by more than 50%]. Post-clinic attendance, the majority of our cases underestimated their risk; this may suggest that false reassurance was derived from the consultation or that women are failing to understand the figures. Alternatively, underestimation may represent a psychological defence and further investigation should attempt to clarify this issue.

Results in terms of adherence to early diagnostic behaviours were of specific interest. Of particular note were the high rates of reported breast self-examination by high-risk women and these fall in line with the existing literature [Kash (1992) similarly reported rates of 90% albeit again practised on an *ad hoc* basis]. Reported breast self-examination by controls was also high [14–40% of general population women have previously been reported to carry out self-examination on a monthly basis (Lauver and Angerame, 1988)]. This was despite there being no consistent advice given at the clinic concerning this practice of unproven benefit. As with our findings, lack of confidence has previously been cited as a barrier to adoption of this secondary prevention technique (Rutledge and Davis, 1988). Despite clinic attendance, 10% of high-risk women reported that they do not perform breast self-examination. A significant minority of women were uncomfortable with examining their breasts, although in this study barriers to breast self-examination were not assessed in those who failed to carry it out. It is notable that the difference between cases and controls in the percentage practising monthly breast self-examination was not large although clinic attenders reported that they had significantly increased their use of this unproven screening method as a result of genetic counselling.

Useful information was gathered in relation to health beliefs surrounding mammography. Results indicate that the vast majority of genetic clinic attenders did not perceive many difficulties in attending for screening. However, only 15% of cases received reminders from their doctors to attend breast screening and such cues have previously been identified as influential in promoting compliance (Vogel *et al.*, 1990). Recent UK reports again suggest that general practitioners and practice nurses may not be taking up valuable opportunities to promote screening participation (Austoker, 1994).

Attitude towards mammography was generally favourable, although a few women felt that mammography exposed them to excessive radiation and a number of women stated that they felt that undergoing a mammogram caused them unnecessary worry. In relation to health beliefs, breast cancer was universally perceived as an extremely serious disease which may be a reflection of ever increasing media attention, high general population incidence rates and greater awareness created by the introduction of a national screening programme.

Generalisation of our findings should be made with caution since the study is retrospective in design and participants were selected from only two cancer family clinics and a single general practice. Many women in our case sample actively sought a referral to the cancer family clinic by approaching their GP for early screening, suggesting a high level of motivation in this sample. Future studies should document source of clinic referral and compare women actively seeking genetic counselling with those attending due to recommendation of a doctor.

*Clinical implications*

Feedback concerning the clinic was generally very favourable, with the vast majority of attenders finding the consultation reassuring, although a significant number still had specific cancer-related worries as indicated by our results. Such clinics may provide an important role in the emotional adjustment of high-risk women who commonly bear a heavy psychological burden from their familial experiences of breast cancer and unusually high bereavement rates. It may be beneficial if clinics can have resources to provide psychological support, with the aim of alleviating excessive breast cancer worry, reducing psychological barriers to secondary prevention, aiding decisions regarding prophylactic surgery and providing counselling for unresolved bereavement reactions.

In terms of educating women about their risk, it is clear that this goal of genetic counselling was only partially attained. There was a failure by two-thirds of those counselled to either comprehend or recall their given risk estimates accurately, despite being sent a follow-up letter summarising the consultation; the benefits of providing annual percentage risk figures is questionable. It may be the 'reassurance' a women receives in the clinic about her chance of breast cancer, or the advice about future screening, that is of primary importance. Clinic attendance appears to have influenced uptake of breast self-examination suggesting that, despite this being an unproven screening method, many high-risk women wish to do something themselves to help them manage their risk of breast cancer. It has also been argued that, since over 90% of breast cancers are found by women themselves, there is a need to optimise this method of detection (Austoker, 1994). One of the most important benefits of genetic counselling is that women attending will subsequently take charge of their risk rather than feel helpless when their high-risk status is confirmed.

*Future research*

The recent cloning of the breast–ovarian cancer susceptibility gene (*BRCA*1), and a second breast cancer susceptibility gene (*BRCA*2), will have a considerable impact on genetic counselling services. Recent estimates suggest that as many as 1 in 800 women may carry a *BRCA*1 mutation (Ford *et al.*, 1995) and potentially a large number of individuals may seek predictive DNA tests (Lerman *et al.*, 1994; Watson *et al.*, 1995). It will become increasingly important to clarify which factors enhance ability to cope with high-risk status, and how best to provide appropriate psychological and medical support. A priority must be to investigate methods of communicating genetic risk information in order to clarify which methods are optimal, and greater account needs to be taken of women's prior expectation and beliefs about genetic inheritance and cancer.

**Acknowledgements**
The study was supported by the Cancer Research Campaign. We are grateful to the following for their contribution to this research: Mrs J Davidson, Mrs T Gladwell, Mrs S Gray, Dr K Kash, Mr M Law, Dr V Murday, Professor B Ponder, Dr M Stratton and to the women who gave their time so freely in answering the study questions.

### References

AUSTOKER J. (1994). Cancer prevention in primary care, screening and self examination for breast cancer. *Br. Med. J.*, **309,** 168–174.

CLAUS EB, RISCH NJ AND THOMPSON WD. (1991). Genetic analysis of breast cancer in the cancer and steroid hormone study. *Am. J. Hum. Genet.*, **48,** 232–242.

DEROGATIS LR AND SPENCER P. (1982). *The Brief Symptom Inventory, Administration, scoring and Procedures Manual*, II. 2nd edition. Clinical Psychometric Research Inc: Baltimore.

EVANS, AM, LOVE RR, MEYEROWITZ BE, LEVENTHAL H AND NERENZ DR. (1985). Factors associated with active participation in a cancer prevention clinic. *Prev. Med.*, **14,** 358–371.

EVANS DGR, BURNELL LD, HOPWOOD P AND HOWELL A, (1993). Perception of risk in women with a family history of breast cancer. *Br. J. Cancer*, **67,** 612–614.

EVANS DGR, BLAIR V, GREENHALGH R, HOPWOOD P AND HOWELL A. (1994). The impact of genetic counselling on risk perception in women with a family history of breast cancer. *Br. J. Cancer*, **70,** 934–938.

FISCHOFF B, BOSTROM A AND QUADREL MJ. (1993). Risk perception and communication. *Annu. Rev. Public Health*, **14,** 183–203.

FORD D, EASTON DF AND PETO J. (1995). Estimates of the gene frequency of *BRCA1* and its contribution to breast and ovarian cancer incidence. *Am. J. Hum. Genet.*, **57,** 1457–1462.

HAILEY BJ. (1991). Family history of breast cancer and screening behaviour: and inverted U-shaped curve? *Med. Hypotheses*, **36,** 397–403.

HOROWITZ M, WILNER N AND ALVAREZ W. (1979). Impact of events scale: a measure of subjective stress. *Psychosom. Med.*, **41,** 209–218.

HOULSTON RS, LEMOINE L, MCCARTER E, HARRINGTON S, MACDERMOT K, HINTON J, BERGER L AND SLACK J. (1992). Screening and genetic counselling for relatives of patients with breast cancer in a family cancer clinic. *J. Med. Genet.*, **29,** 691–694.

JANIS IL AND FESCHBACH S. (1953). Fear arousing communications. *J. Abnorm. Psychol.*, **48,** 78–92.

JANZ NK AND BECKER MH. (1984). The health belief model: a decade later. *Health Educ. Q.*, **11,** 1–47.

JOSTEN DM, EVANS AM AND LOVE RR. (1986). The cancer prevention clinic: a service program for cancer-prone families. *J. Psychosoc. Oncol.*, **3,** 5–20.

KASH KM, HOLLAND JC, HALPER MS AND MILLER DG. (1992). Psychological distress and surveillance behaviours of women with a family history of breast cancer. *J. Natl. Cancer Inst.*, **84,** 24–30

KELLY PT (1983). Counselling persons who have family histories of cancer. In *The Practice of Cancer Prevention in Clinical Medicine*, Newell GR (ed.) Raven Press: New York.

LAUVER D AND ANGERAME M. (1988). Development of a questionnaire to measure beliefs and attitudes about breast self-examination. *Cancer Nurs.*, **11,** 51–57.

LERMAN C, DALY M, SANDS C, BALSHEM A, LUSTBADER E, HEGGAN T, GOLDSTEIN L, JAMES J AND ENGSTROM P. (1993). Mammography adherence and psychological distress among women at risk for breast cancer. *J. Natl Cancer Inst.*, **85,** 1074–1080.

LERMAN C, DALY M, MASNY A AND BALSHEM A. (1994). Attitudes about genetic testing for breast ovarian cancer susceptibility. *J. Clin. Oncol.*, **12,** 843–850.

MIKI Y, SWENSEN J, SHATTUCK-EIDENS D, FUTREAL A, HARSHMAN K, TAVTIGIAN S, QINGYUN L, COCHAN C, BENNETT LM, DING W, BELL R, ROSENTHAL J, HUSSEY C, TRAN T, MCCLURE M, FRYE C, HATTIER T, PHELPS R, HAUGEN-STRANO A, KATCHER H, YAKUMO K, GHOLAMI Z, SHAFFER D, STONE S , BAYER S, WRAY C, BOGDEN R, DAYANANTH P, WARD J, TONIN P, NAROD S, BRISTOW PK, NORRIS FH, HELVERING L, MORRISON P, ROSTECK P, LAI M, BARRETT JC, LEWIS C, NEUHAUSEN S, CANNON-ALBRIGHT L, GOLDGAR D, WISEMAN R, KAMB A AND SKOLNICK MH. (1994). A strong candidate for the breast and ovarian cancer susceptibility gene BRCA1. *Science*, **266,** 66–71.

RUTLEDGE DN AND DAVIS DT. (1988). Breast self-examination, compliance and the health belief model. *Oncol. Nurs. Forum*, **15,** 175–179.

SHILOH S AND SAGI M. (1989). Effect of framing on the perception of genetic recurrence risks. *Am. J. Med. Genet.*, **33,** 130–135.

STEEL M, THOMPSON A AND CLAYTON J. (1991). Genetic aspects of breast cancer. *Br. Med. Bull.*, **47,** 504–518.

VOGEL VG, SCHREIBER GRAVES D, VERNON SW, LORD JA, WINN RJ AND PETERS GN. (1990). Mammographic screening of woman with increased risk of breast cancer. *Cancer*, **66,** 1613–1620.

WATSON M, MURDAY V, LLOYD S, PONDER P, AVERILL D AND EELES R. (1995). Genetic testing in breast/ovarian cancer (*BRCA*1) families. *Lancet*, **346,** 583.

WELLISCH DK, GRITZ ER, SCHAIN W, WANG H AND SIAU J. (1991). Psychological functioning of daughters of breast cancer patients, part 1: daughters and comparison subjects. *Psychosomatics*, **32,** 324–336.

WELLISCH DK, GRITZ ER, SCHAIN W, WANG H AND SIAU J. (1992). Psychological functioning of daughters of breast cancer patients, part 11: characterizing the distressed daughter of the breast cancer patient. *Psychosomatics*, **33,** 171–179.

WOOSTER R, BIGNELL G, LANCASTER J, SWIFT S, SEAL S, MANGION J, COLLINS N, GREGORY S, GUMBS C, MICKLEM G, BARFOOT R, HAMOUDI R, PATEL S, RICE C, BIGGS P, HASHIM Y, SMITH A, CONNOR F, ARASON A, GUDMUNDSSON J, FICENEC D, KELSELL D, FORD D, TONIN P, BISHOP D, SPURR N, PONDER B, EELES R, PETO J, DEVILEE P, CORNELISSE C, LYNCH H, NAROD S, LENOIR G, EGILSSON V, BARKADOTTIR R, EASTON D, BENTLEY D, FUTREAL P, ASHWORTH A AND STRATTON M. (1995). Identification of the breast cancer susceptibility gene BRCA2. *Nature*, **378,** 789–792.